# UK guidelines for the management of bone sarcomas

**DOI:** 10.1038/s41416-024-02868-4

**Published:** 2024-11-16

**Authors:** Craig Gerrand, Fernanda Amary, Hanny A. Anwar, Bernadette Brennan, Palma Dileo, Maninder Singh Kalkat, Martin G. McCabe, Anna Louise McCullough, Michael C. Parry, Anish Patel, Beatrice M. Seddon, Jennifer M. Sherriff, Roberto Tirabosco, Sandra J. Strauss

**Affiliations:** 1 Bone and Soft Tissue Tumour Service, Royal National Orthopaedic Hospital, Stanmore, Middlesex HA7 4LP UK; 2 The Christie NHS Foundation Trust, Manchester, M20 4BX UK; 3 Department of Oncology, University College London Hospital NHS Foundation Trust, London, NW1 2BU UK; 4 Department of Thoracic Surgery, Queen Elizabeth Hospital, Birmingham, B15 2GW UK; 5 Department of Orthopaedics, Woodend Hospital, Eday Road, Aberdeen, AB15 6XS UK; 6 Royal Orthopaedic Hospital, Bristol Road South, Birmingham, B31 2AP UK; 7 Cancer Centre, Queen Elizabeth Hospital, Birmingham, UK

**Keywords:** Sarcoma, Bone cancer

## Abstract

This document is an update of the British Sarcoma Group guidelines (2016) and provides a reference standard for the clinical care of UK patients with primary malignant bone tumours (PMBT) and giant cell tumours (GCTB) of bone. The guidelines recommend treatments that are effective and should be available in the UK, and support decisions about management and service delivery. The document represents a consensus amongst British Sarcoma Group members in 2024. Key recommendations are that bone pain, or a palpable mass should always lead to further investigation and that patients with clinical or radiological findings suggestive of a primary bone tumour at any anatomic site should be referred to a specialist centre and managed by an accredited bone sarcoma multidisciplinary team. Treatment recommendations are provided for the major tumour types and for localised, metastatic and recurrent disease. Follow-up schedules are suggested.

## Introduction

### Rationale and objective of guidelines

Bone sarcomas are rare and require centralised management. NHS England commissions the diagnosis and surgical treatment of primary malignant bone tumours (PMBT) in five designated highly specialised bone sarcoma centres or where this is not possible, according to pathways agreed by bone tumour MDTs. Chemotherapy and radiotherapy are delivered in commissioned oncology centres as described in the 2019 service specification [1] and this will likely continue under the 2021 Health and Care Bill. Scotland has a designated Sarcoma Network and patients in Northern Ireland may be referred elsewhere in the United Kingdom (UK). Patients from Wales may have surgery in specialist centres in England, with other treatments in Wales.

This update of the 2016 British Sarcoma Group (BSG) guidelines [2] aims to improve the quality of care for patients with bone tumours in the UK by supporting key management decisions. They represent a consensus in 2024 but will require updates as treatment evolves.

### Scope

These guidelines apply to all types of PMBT, including giant cell tumours of bone (GCTB), arising in any skeletal location. They recommend effective treatments which should be available to UK bone sarcoma multidisciplinary teams (MDT). Haemopoietic tumours of bone, rehabilitation, prosthetic services, and palliative care are not included.

### Methods

The following were consulted: NCCN Clinical Practice Guidelines Version 2.2022 [3]; Bone sarcomas: ESMO-EURACAN-GENTURIS-ERN PaedCan Clinical Practice Guideline for diagnosis, treatment and follow-up [4]; National Institute for Health and Care Excellence (NICE) Sarcoma NICE Quality Standard QS78 [5]; and Suspected cancer: diagnosis and referral guideline [6] as well as published literature from 2010 to 2023. The authors considered applicability to UK practice and reached a consensus about the content. The document was then available to BSG members for review.

## Incidence and epidemiology

PMBTs comprise 0.2% of all cancers with an annual incidence of around 8.5 per million in Europe [7]. On average, around 580 people are diagnosed with PMBT or GCTB of bone each year in England. Therefore, a UK General Practitioner (GP) is unlikely to see such a patient in a working lifetime. Diagnostic delays are common and may lead to poorer outcomes.

PMBTs comprise 5% of childhood cancers in Europe: the two major diagnoses are osteosarcoma and Ewing sarcoma [8]. In children under 5 years, metastatic neuroblastoma or eosinophilic granuloma are more common. Chondrosarcoma is more common in older patients [9].

In adults, especially over 40 years, metastatic carcinomas (usually bronchus, breast, thyroid, kidney or prostate) and haemopoietic malignancies (e.g. plasma cell tumour or lymphoma) in bone considerably outnumber PMBT. At any age benign lesions or infections are possible. If there is diagnostic uncertainty, a PMBT must be excluded.

There is variation in survival across Europe: 5-year survival for patients in Ireland and the UK is lower than in Northern and Central Europe (52.4% vs 62.6 and 61.7% respectively) [10].

## Classification of bone sarcomas

Primary malignant bone tumours are in general classified morphologically (Table 1).

**Table 1:** Classification of malignant primary bone tumours (adapted from WHO 2020) [19].

Chondrogenic tumours	1. Atypical cartilaginous tumour/Chondrosarcoma grade 1 2. Chondrosarcoma (grades 2–3) 3. Periosteal chondrosarcoma 4. Clear cell chondrosarcoma 5. Mesenchymal chondrosarcoma 6. Dedifferentiated chondrosarcoma
Osteogenic tumours	1. Low-grade central osteosarcoma 2. Osteosarcoma NOS (conventional osteosarcoma, telangiectatic osteosarcoma, small cell osteosarcoma) 3. Parosteal osteosarcoma 4. Periosteal osteosarcoma 5. High grade surface osteosarcoma 6. Secondary osteosarcoma
Fibrogenic tumours	1. Fibrosarcoma NOS
Vascular tumours	1. Epithelioid haemangioendothelioma NOS 2. Angiosarcoma
Osteoclastic giant-cell rich tumours	1. Giant cell tumour of bone, malignant
Notochordal tumours	1. Chordoma NOS (Chondroid chordoma) 2. Poorly differentiated chordoma 3. Dedifferentiated chordoma
Other mesenchymal tumours of bone	1. Adamantinoma of long bones (Dedifferentiated adamantinoma) 2. Leiomyosarcoma NOS 3. Pleomorphic sarcoma, undifferentiated 4. Ewing sarcoma 5. Round cell sarcoma with *EWSR1*::non-*ETS* fusions 6. *CIC*-rearranged sarcoma 7. Sarcoma with *BCOR* genetic alterations

### Chondrosarcoma

Chondrosarcoma most commonly presents between 30 and 60 years. Demographic changes mean chondrosarcoma has become the most common PMBT [9, 11].

Differentiating between enchondroma and low-grade chondrosarcoma can be difficult. The WHO classifies these as atypical cartilaginous tumours in the appendicular skeleton, and grade 1 chondrosarcoma in the axial skeleton (including scapula, pelvis and skull). Central atypical cartilaginous tumour is of intermediate malignancy, usually locally aggressive and rarely metastasising. Care must be taken to avoid the overtreatment of benign tumours and the undertreatment of malignant ones [12].

Most chondrosarcomas arise in long bones but can also arise in flat bones (eg pelvis, rib and scapula). Chondrosarcomas arising in pre-existing benign osteochondromas and enchondromas are termed secondary peripheral chondrosarcoma and secondary central chondrosarcoma respectively. The risk of developing chondrosarcoma in solitary osteochondromas and enchondromas is uncertain but increased with multiple lesions or in the axial skeleton, particularly the pelvis [13].

Most primary chondrosarcomas are low- rather than high-grade [14]. Rarer types include mesenchymal and clear-cell chondrosarcoma. Rarely, conventional chondrosarcomas become very high-grade dedifferentiated chondrosarcomas with a poor prognosis [15, 16].

Extraskeletal myxoid chondrosarcoma is considered a soft tissue sarcoma.

### Osteosarcoma

Osteosarcoma is the second most frequent primary bone cancer, comprising 4% of solid cancers in children and 3% in teenagers and young adults (TYA). A second peak occurs in the seventh and eighth decades. It is slightly more common in males (1.4:1) and black patients. Survival rates are higher in younger patients [9, 17].

Osteosarcoma includes high-grade conventional, intermediate- and low-grade tumours (e.g. low-grade central and parosteal) variants. The latter can have high-grade components.

Osteosarcoma usually arises in the metaphysis of extremity long bones, most commonly around the knee. Some tumours (predominantly in adults) arise in the axial skeleton, pelvis or craniofacial bones. Risk factors for osteosarcoma include previous radiation therapy, Paget’s disease of bone and germ-line abnormalities such as Li-Fraumeni syndrome, Werner syndrome, Rothmund -Thomson syndrome and familial retinoblastoma [18].

### Ewing sarcoma

Ewing sarcoma is the second most common primary malignant bone tumour in children and adolescents but can occur in adults. Median age at diagnosis is around 15 years. It is more common in males (1.5:1) and less common in people of Chinese or Black African origin [9].

The most frequent sites are long bones, pelvis, ribs, and vertebral column. All are high grade. Ewing sarcoma is characterised by rearrangements between members of the FET (also known as TET) family genes, most commonly EWSR1 located on chromosome 22, and ETS family genes, most commonly FLI1 located on chromosome 11. EWSR1::FLI1 and other FET::ETS gene fusions are pathognomonic in context with morphology [19].

### Round cell sarcoma with *EWSR1*:non-ETS fusions and other Ewing-like sarcomas

These newly defined entities were previously called Ewing-like sarcomas. Some retain EWSR1 as a gene fusion partner, including EWSR1::NFATC2, EWSR1::PATZ1. Others include non-FET::non-ETS fusions, including CIC-rearranged and BCOR altered sarcomas which can arise in bone or soft tissue. Although frequently treated in the same way as ES, CIC-rearranged sarcomas have a poorer response to chemotherapy and an unfavourable prognosis [20, 21].

### Undifferentiated pleomorphic sarcoma of bone

Undifferentiated pleomorphic sarcomas of bone (UPS, previously called malignant fibrous histiocytoma) have no specific differentiation. They are typically high-grade with metastatic rates of at least 50%. Treatment usually involves neoadjuvant therapy followed by wide excision. They show similar chemosensitivity and survival rates to osteosarcoma [22]. Occasionally, UPS is found to be a dedifferentiated chondrosarcoma or osteosarcoma after resection.

### Chordoma

Chordomas develop from persistent notochordal elements: nuclear expression of brachyury is diagnostic [23]. They originate from the sacrum (50%), skull base (30%), and mobile spine (20%). Extraskeletal tumours are very rare. The current World Health Organisation (WHO) classification divides chordoma into three subtypes the most common of which is conventional chordoma which is locally invasive and typically low-grade. Metastases occur in 30–40% of patients, typically late and usually after local recurrence. Metastases can occur in lung, liver, bone, sub-cutis, lymph nodes and other sites [24]. Dedifferentiated and poorly differentiated chordoma (PDC) are rare, aggressive subtypes.

### Adamantinoma

Adamantinoma is a rare, low-grade malignant neoplasm arising in the tibia, fibula or both bones and rarely in other bones [25]. Adamantinoma accounts for 0.3% to 1% of all PMBT and occurs mostly in young to middle-aged adults (20 to 40 years of age), with a male-to-female ratio of 1.3:1. The tibial shaft (medial or distal), is most commonly affected. There are lytic and sometimes destructive areas which can lead to fracture [26]. Recurrence is late (can be >20 years) but frequent (about 30%) after incomplete excision. The metastatic rate is 10% to 20%, usually lung [25].

### Giant cell tumour of bone

GCTBs are relatively rare, representing 12–15% of primary bone tumours in England. Incidence is higher in Asia [27]. GCTBs usually occur between 20 and 40 years of age and are rare before epiphyseal closure. Tumours usually occur at the epiphyses of long bones next to joints but may arise in other bones, and are rarely multicentric. Histologically tumours comprise mononuclear stromal cells with numerous scattered multinucleated giant cells (osteoclasts) recruited by rank-ligand from stromal cells.

GCTBs are usually locally aggressive and may demonstrate a soft tissue mass or pathological fracture. There is a high risk of local recurrence (up to 50%) and approximately 5% metastasise to the lungs, particularly after local recurrence [28–30]. Both conventional and malignant GCTs demonstrate a mutation in the H3F3A gene, the detection of which can help diagnosis, especially distinguishing from giant-cell enriched osteosarcoma [30, 31].

### Other malignant mesenchymal tumours

Very rarely (between 2% and 5% of PMBTs) malignant spindle cell mesenchymal tumours which usually occur in soft tissues (eg leiomyosarcoma, fibrosarcoma) present as a PMBT [32]. These arise in a similar age group to chondrosarcoma, but the skeletal distribution is closer to osteosarcoma. There is a high incidence of fracture at presentation. Associations with pre-existing conditions (eg Paget’s disease or bone infarct) or previous irradiation have been reported [33].

## Presentation and referral

The most common symptoms of PMBT are pain and/or a lump or swelling [34]. Some patients present with a pathological fracture. Bone pain may vary in intensity, but night pain is a ‘red flag’ requiring further investigation. A mass may develop later. Patients with spinal tumours may present with spinal cord compression or other neurological symptoms. Systemic symptoms are unusual but may indicate metastatic disease. The average duration of symptoms is 3 months, but many patients present later. A history of recent injury does not exclude PMBT.

Assessment should comprise a full history (including duration, intensity and diurnal variation of pain, prior benign or malignant tumours, family history and previous radiotherapy). Clinical examination should consider the size, consistency, mobility, and location in relation to bone of any mass, regional and local lymph nodes, adjacent joints and neurovascular examination.

An urgent x-ray of the affected site is required [6]. Further investigation is needed if the X-ray shows any of:

- Bone destruction
- New bone formation
- Periosteal swelling
- Soft tissue swelling

A ‘normal’ x-ray does not exclude PMBT. Persistent pain or a mass requires an urgent magnetic resonance imaging (MRI) scan or referral to a bone sarcoma centre.

Patients under 40 years with suspected PMBT should be referred urgently to a bone sarcoma centre. If possible, investigations before referral should include an X-ray of the affected site and blood tests including full blood count (FBC), erythrocyte sedimentation rate (ESR), and biochemical profile with alkaline phosphatase (ALP)). Local site imaging (ideally MRI) before referral is appropriate if there is minimal delay.

In patients over 40 years metastatic carcinoma in bone is likely and prompt investigation before referral is appropriate. Suitable investigations include CT of the chest, abdomen and pelvis, whole skeletal imaging (eg isotope bone scan or whole-body MRI), and myeloma screen. Patients with proven solitary bone lesions should be referred to a bone sarcoma centre to exclude PMBT.

Patients with suspected PMBT should be referred to a commissioned bone sarcoma centre on an urgent cancer pathway [6]. Patients should be referred before biopsy because poorly performed biopsies can compromise treatment [35, 36] and specialist pathology is required. All histological diagnoses of PMBT should be reviewed by a specialist pathologist within a bone sarcoma MDT.

Networks should ensure GPs and hospital doctors are aware of the diagnostic pathways for PMBTs and comply with the NICE Suspected cancer: diagnosis and referral guideline [6]. Referral guidelines for Scotland are on the NHS Scotland website (https://www.cancerreferral.scot.nhs.uk/sarcomas-and-bone-cancers/) [37].

### Key recommendations

- The most common symptoms of PMBT are a mass or pain. Bone pain at night is a ‘red flag’ symptom requiring investigation.
- Plain X-ray is the first investigation of choice.
- Radiological features including bone destruction, new bone formation, periosteal swelling and/or soft tissue swelling require further investigation.
- A normal X-ray does not exclude PMBT, and an urgent MRI may be required.
- Networks should ensure GPs and hospital doctors are aware of urgent referral criteria and diagnostic pathways for PMBT.
- All provisional histological and/or radiological diagnoses of PMBT should be reviewed by a specialist sarcoma pathologist and/or radiologist, within a bone sarcoma MDT.

## Investigation

### Imaging

All patients with PMBT should have X-rays in two planes.

MRI of the local site including the whole anatomical compartment, the whole of the involved bone and adjacent joints is required. CT is helpful if there is diagnostic uncertainty or MRI is contraindicated and may demonstrate microcalcification, periosteal bone formation and cortical destruction. CT and MRI are routine for pelvic tumours. Dynamic contrast-enhanced MRI may identify high-grade areas within a chondrosarcoma, guiding biopsy [38].

Patients with confirmed PMBTs should have staging investigations including chest CT. Indeterminate nodules in the lungs usually require an interval scan. All suspicious chest CTs should be reported by a bone sarcoma MDT radiologist.

Whole skeleton, and ideally whole-body imaging, is required in Ewing sarcoma and osteosarcoma. Whole-body MRI and positron emission tomography (PET)CT are replacing bone scintigraphy for staging and treatment response evaluation [39–41]. Bone marrow biopsies are no longer mandated outside of clinical trials [42]. Skeletal metastases are rare in chondrosarcoma, unless the tumour is dedifferentiated [43].

During chemotherapy clinical assessment (pain and clinical measurement) and appropriate imaging of the local site (e.g. by MRI, CT or PET-CT) and lungs (CT chest) are helpful to evaluate chemotherapy response [44, 45].

### Staging systems

Two staging systems are used. The Enneking system uses histological grade (I = low and II = high grade) and extent in relation to anatomical compartments (A = intracompartmental, B = extracompartmental). If the bone cortex is intact and there is no soft tissue mass, the tumour is intracompartmental. Stage III tumours have metastases but can be high or low-grade [46].

The TNM system (American Joint Committee on Cancer – AJCC/International Union against cancer - UICC) is based on tumour grade, size and the presence of metastases [47].

### Laboratory tests

There are no specific laboratory tests for the diagnosis of PMBT. However, ESR, alkaline phosphatase (ALP) and lactate dehydrogenase (LDH) may be of prognostic value [48, 49].

### Other baseline assessments

Around 10% of Ewing sarcomas metastasise to bone marrow. Whole body MRI and/or CT PET scan are less invasive and give equivalent information to bone marrow (BM) sampling [50] which is therefore no longer mandated.

Pre-chemotherapy evaluation should include renal function (e.g. urea, creatinine, glomerular filtration rate) and cardiac function (e.g. echocardiogram, MUGA [multi-gated acquisition scan]). An audiogram is recommended before cisplatin.

### Fertility preservation

Sperm storage is recommended for male patients of reproductive age. All female patients of reproductive age should be referred for fertility counselling, delivered as early as possible in the treatment planning process [51, 52] and should be within current ASCO guidelines [53]. Options include hormone or egg-harvesting if time is sufficient time prior to the commencement of chemotherapy. Ovarian cryopreservation remains experimental.

### Biopsy

Biopsy is the definitive diagnostic test for PMBT and should be performed at a bone sarcoma centre in consultation with the surgical team to provide access to molecular diagnostic techniques and ensure the biopsy track can be excised [35]. Poor biopsies can compromise limb salvage or even cure. The principles of biopsy are:

- Biopsy should only be done after cross-sectional imaging to identify the best area to biopsy and plan the approach.
- Minimal contamination of normal tissues.
- Core needle biopsy has a high diagnostic yield but may require a general anaesthetic and is ideally guided by ultrasound, X-ray or CT.
- Samples should be taken for microbiology, histology and cytogenetic/molecular genetic studies including whole genome sequencing.
- Where possible, samples should be snap-frozen in a tumour bank for future research with patient consent.
- Samples must be evaluated by a specialist pathologist who is a core member of a bone sarcoma MDT.
- The pathology request form should include clinical information, including anatomical site, age and the radiological differential diagnosis.

Image-guided core needle biopsy is safe and has a high diagnostic yield for Ewing sarcoma [54] but is less accurate in identifying the grade of chondral tumours [55]. Assessing the grade of chondrosarcomas is difficult and opinions often vary [14]. Diagnosis of chondrosarcoma requires discussion in a bone sarcoma MDT.

CT-guided biopsies may be better for deeper locations (e.g. pelvis) or to target a particular area within a tumour (e.g. a dedifferentiated area in a chondrosarcoma) [56, 57]. Frozen sections can confirm lesional tissue is present but are unreliable for definitive diagnosis and consume diagnostic material. Biopsy tracks should be clearly marked with a small incision or tattoo to guide later excision.

Biopsy of possible metastases, including locoregional lymph node spread, should be considered before treatment starts if management might change as a result (e.g. trial entry, decision to amputate or inclusion in the radiation field).

‘Liquid biopsies’ investigating circulating free (cf) or circulating tumour (ct) DNA are emerging as possible diagnostic tools. Exploratory studies have shown that cfDNA can be discriminated between sarcoma and non-sarcoma patients, and between Ewing and non-Ewing sarcomas, and serial ctDNA tests may reflect response and relapse in Ewing sarcoma, but these do not replace histological diagnosis [58, 59].

Laminectomy or decompression for potential spinal PMBTs should be avoided at diagnosis unless necessary for emergency relief of spinal cord compression (SCC) and only after consultation with a member of a bone sarcoma MDT. Some patients with impending or symptomatic SCC remain neurologically stable with non-surgical treatment granting time for histological diagnosis to be obtained and definitive management to be planned at a bone sarcoma centre. Emergency intralesional decompression usually prevents later complete removal of a PMBT.

### Pathology

Pathologists reporting biopsies and/or resections of bone sarcomas should be accredited bone tumour pathologists and members of a bone sarcoma MDT.

Reports should comply with Royal College of Pathologists guidance and [60] according to recommendations from the International Collaboration on Cancer Reporting (ICCR) where possible [61].

Reports of definitive resections should include gross descriptions of the location and size (measured in three dimensions in mm) of the tumour, the extent of local tumour spread and the involvement of specific anatomical compartments. Resection margins should be reported as clear or involved. The distance (in mm) of the tumour from the nearest resection margin and the nature of tissue at this margin should be specified as well as the estimated response to treatment if appropriate.

Histological features of the tumour and further investigations (e.g. immunohistochemistry or molecular genetics) should be recorded. The tumour type (and subtype) should be recorded using WHO criteria [19]. The tumour type should be coded using Systematised Nomenclature of Medicine – Clinical Terms (SNOMED-CT) codes [62].

### Molecular genetics and pathology

Tissue banks are essential for diagnostic and translational research, therefore informed consent for tumour banking, analysis and research should be sought and fresh frozen tissue stored whenever possible. Patients should have access to whole genome sequencing as part of routine care [63].

Although most Ewing sarcomas are recognised morphologically and by immunohistochemical identification of the surface glycoprotein CD99, molecular genetic confirmation of the Ewing sarcoma translocation is mandated. Detection of *EWSR1* gene rearrangement is required either by fluorescence in situ hybridisation (FISH) and/or by identification of the specific FET::ETS fusion. The latter is available as part of the RNA panels within the Genomic Laboratory Hubs.

Molecular analysis will also diagnose other rare round cell sarcomas, such as CIC-rearranged and BCOR-altered sarcomas and other newer entities [19].

### Confirmation of diagnosis

The diagnosis and management of all patients with suspected PMBT should be discussed in a bone sarcoma MDT with a surgeon, radiologist, pathologist and oncologist with access to all clinical information and biopsy material [35, 64].

### Key recommendations

- Patients with suspected PMBT should have access to timely and appropriate imaging.
- The definitive diagnostic test is biopsy, performed at a Specialist Sarcoma Centre by an expert radiological or surgical team considering the definitive tumour resection.
- All patients should have tissue stored with appropriate consent and should have access to whole genome sequencing where possible.
- Diagnostic and resection specimens should be examined by an accredited bone tumour pathologist who is part of a bone sarcoma MDT and should comply with the Royal College of Pathologists guidance.
- The diagnosis must be confirmed by reference to clinical findings, laboratory investigation and radiological imaging at a bone sarcoma MDT.
- Patients with a confirmed diagnosis should be staged according to AJCC criteria.
- If treatment may affect fertility, patients should be referred to the appropriate reproductive medicine service before commencing treatment.

## Overview of management

Patients should have a key worker as well as a specialised MDT [35]. Children, teenagers and young adults should also be discussed at the relevant children’s or TYA (teenage and young adult) MDT. There should be sufficient specialist staff to ensure age-appropriate care.

Bone sarcoma MDTs should be properly constituted, with sufficient core members of the relevant specialities, and meet the criteria for the minimum number of patients treated each year; they should collect data on patients, tumours, treatment and outcomes as agreed nationally and participate in national audit [64].

Patients should be supported to participate in clinical trials. Information about trials is available on the Sarcoma UK website (https://sarcoma.org.uk/clinical-trials-hub/).

### Chemotherapy

Chemotherapy is a standard treatment for most osteosarcoma, Ewing sarcoma, other round-cell sarcomas, and high-grade sarcomas of bone. Chondrosarcoma is usually treated surgically with chemotherapy considered in dedifferentiated and mesenchymal variants.

The usual sequence of treatment is neoadjuvant combination chemotherapy, local treatment (surgery and/or radiotherapy) and post-operative adjuvant chemotherapy. The main aim of chemotherapy is to decrease the incidence of distant relapses, but neoadjuvant chemotherapy may help control the primary tumour.

### Surgery

Surgery is usually essential for cure and may be the only treatment required. Surgery should be part of a multidisciplinary treatment plan and be performed by a core member of a bone sarcoma MDT in a commissioned centre, or where this is not possible (for example because specialist site-specific surgical expertise is required) in consultation with a bone sarcoma MDT.

Decisions about the timing of surgery and the most appropriate procedure (eg limb salvage or amputation) should be agreed in the MDT. The MDT should consider tumour size, involvement of anatomical structures, response to neoadjuvant treatments, patient preferences and whether complete removal is possible. Decisions about surgical reconstruction should follow discussion of the risks and benefits of available options, the expected functional outcomes and patient and surgeon preferences. Surgeons should be familiar with a range of reconstructive techniques, including for children and adolescents.

The aim of surgery should be to remove the whole tumour. This means en-bloc resection of involved parts of bone and soft tissue with adequate margins, and in Ewing sarcoma surgeons should consider including all structures involved with the tumour volume at presentation. It may be helpful to mark close surgical margins with (MRI-inert) haemostatic clips in the surgical field. Resection specimens should be orientated (for example with sutures) to allow the location and thickness of surgical margins to be reported.

Surgery for PMBT is often complex, requiring a surgical team with appropriate skills. For example, surgery for chest wall PMBT may involve the removal of ribs, sternum, vertebra and overlying muscles and skin. The resulting chest wall defect may interfere with respiration, risk injury of vital structures and require reconstruction (for example with mesh, cement and/or titanium) as well as soft tissue coverage. This requires a multidisciplinary surgical team including thoracic, spinal and plastic surgeons. In addition, appropriate pre-rehabilitation, involvement in ERAS (Enhanced recovery after surgery) and intensive physiotherapy in the post-operative period is required [65, 66].

Surgery for local recurrences or metastatic disease requires discussion in a bone sarcoma MDT.

### Requirements for the surgical report

Operation records should describe the procedure, the tissues resected and areas where the resection was close to or involved the tumour mass. Bone and soft tissue reconstructions should be described as well as the use of prophylactic antibiotics and thromboprophylaxis (e.g. mechanical and/or chemical agents). Post-operative care and rehabilitation should be clearly communicated to all members of the MDT [67].

### Radiotherapy

Radiotherapy is used selectively for PMBT. In Ewing sarcoma, It can be used as a definitive treatment for the primary tumour, or in combination with surgery either pre-operatively or post-operatively [68, 69]. The role of radiotherapy is much less for non-Ewing primary bone sarcomas (including osteosarcoma and chondrosarcoma), it is used as a definitive treatment only if surgery is not possible, and post-operatively in selected cases considered to be at particularly high risk of local recurrence [70]. Radiotherapy has a palliative role in all tumour types.

Particle therapy, proton beam therapy (PBT) or carbon ion radiotherapy (CIRT) may be effective in the treatment of unresectable primary bone sarcomas [71, 72]. Potential advantages of particle therapy over intensity-modulated radiotherapy (IMRT) include dose escalation near critical structures and reduced late toxicity including radiation-induced malignancy, which is particularly important for children and teenagers and young adults.

In skull base chondrosarcomas or chordomas, surgery and PBT can achieve local control rates of approximately 70–90% [73–75]. Similarly, high local control rates have been reported in sacral chordomas with definitive PBT or CIRT [76–78] or in the post-operative setting [79]. A retrospective study of PBT for incompletely resected or unresectable osteosarcoma reported a five-year DFS of 65%, and a five-year OS of 67% [72].

Currently, there are two UK NHS proton facilities, at the Christie NHS Foundation Trust in Manchester and University College London Hospital NHS Foundation Trust.

### Prevention and management of pathological fracture

Patients with an impending or completed pathological fracture from a suspected PMBT require external splintage or immobilisation and pain control until a diagnosis is established after imaging and biopsy. Internal fixation is contraindicated.

Although pathological fracture is associated with poorer survival and higher local recurrence in osteosarcoma [80] limb-sparing surgery may be possible [81]. Fractures often heal during neoadjuvant chemotherapy and resection of the tumour and involved soft tissues can follow. Amputation may still be indicated if there is no radiological response and/or resection of the tumour and contaminated structures will not safely leave a useful limb. Adjuvant radiotherapy may decrease the risk of local recurrence in osteosarcoma and may have a role in other tumours after pathological fracture but at the risk of complications of limb reconstruction.

Pathological fracture in chondrosarcoma may indicate higher tumour grade [82], and in localised dedifferentiated chondrosarcoma amputation may offer better local control rates [83].

### Pulmonary metastatectomy

Pulmonary metastatectomy may be indicated for oligometastatic disease, aiming to remove all lesions seen on high-resolution CT scans while preserving healthy lung tissue. Thoracotomy is traditionally preferred but there is no clear advantage over thoracoscopic resection [84].

Resection of suspicious pulmonary lesions after primary treatment and control of the primary site should be considered for diagnosis and prognosis. A good prognosis is indicated by longer disease-free intervals, fewer stable lesions, and favourable histology. Advanced imaging and minimally invasive techniques such as video or robotic-assisted thoracoscopic surgery and radiofrequency ablation may improve recovery times and satisfactory outcomes.

### Key recommendations

- All patients with a confirmed PMBT should have care supervised by a bone sarcoma MDT and be allocated a key worker. Children, teenagers and young adults should also be discussed at the relevant children’s or TYA (young adult) MDT.
- Networks should ensure the needs of children and young people with cancer are met with sufficient specialist staff and age-appropriate care and facilities.
- A bone sarcoma MDT should meet the minimum criteria for the number of patients treated and requirements for core membership of the relevant specialities.
- All bone sarcoma MDTs should collect data on patients, tumours, treatment and outcomes as agreed nationally.
- Patients should undergo definitive resection of their sarcoma by a surgeon who is a member of a bone sarcoma MDT in a commissioned centre or if more appropriate, by a surgeon with tumour site-specific or age-appropriate skills, in consultation with the bone sarcoma MDT.
- When considering the local treatment of bone tumours, options for amputation or limb-sparing surgery should be tailored to the preferences of the patient.
- Chemotherapy and radiotherapy should be carried out at designated centres by appropriate specialists as recommended by a bone sarcoma MDT.
- The bone sarcoma MDT should consider referring patients with pulmonary metastases to a thoracic surgeon to consider pulmonary metastatectomy.

### Specific treatment

#### Chondrosarcoma

Surgery is the treatment of choice for most chondrosarcomas. Prognostic factors for conventional chondrosarcoma include metastatic disease at presentation, histological grade, axial primary site and size [85]. Metastatic disease at presentation is more common in dedifferentiated and mesenchymal chondrosarcoma [86].

In the extremity ACTs can be managed by observation initially [87]. If there is progression or symptoms, complete curettage with or without surgical adjuvants (e.g. high-speed burr, cryotherapy) has a high chance of local control. However, grade progression may occur after local recurrence and excision may be preferred.

Low-grade peripheral chondrosarcomas should be completely removed, aiming to leave a covering of normal tissue.

Higher-grade chondrosarcomas (including clear cell chondrosarcoma) and all chondrosarcomas of the pelvis or axial skeleton should be surgically removed with wide margins [15, 16]. In the pelvis a 2 mm margin is associated with lower local recurrence rates [88].

In dedifferentiated chondrosarcoma, complete excision is recommended if feasible. There is a high risk of local recurrence following pathological fracture. Amputation reduces the risk of local recurrence if wide margins cannot be achieved but there is a high risk of metastasis [83].

Patients with multiple osteochondromas or multiple enchondromas (Ollier or Mafucci disease) are at risk of developing secondary chondrosarcomas and should be counselled and followed up appropriately.

#### Radiotherapy

Radiotherapy may be offered for unresectable disease, adjuvantly after surgery for close or positive margins, and for palliation. Particle therapy (PBT or CIRT) for tumours close to critical normal structures or stereotactic techniques for smaller tumours may be helpful to allow dose escalation [89].

High-dose radiotherapy is recommended for skull base chondrosarcomas and with surgery can achieve high (80–90%) local control rates [90].

#### Chemotherapy

Patients with mesenchymal chondrosarcoma may be considered for adjuvant or neoadjuvant chemotherapy [91, 92].

Although the role of chemotherapy in dedifferentiated chondrosarcoma is not well defined, osteosarcoma chemotherapy protocols can be considered as neo-adjuvant and or adjuvant therapy although survival is unfortunately poor [93].

#### Recurrent and metastatic disease

Local recurrence of chondrosarcoma is best treated by further wide excision [94]. Inoperable, locally advanced and metastatic chondrosarcomas have a poor prognosis [95]. Surgery or local ablation should be considered for oligometastatic pulmonary disease.

Chemotherapy is of limited benefit in metastatic mesenchymal or dedifferentiated chondrosarcoma. Preliminary data supports the use of trabectedin in mesenchymal chondrosarcoma. Pazopanib has demonstrated activity in conventional chondrosarcoma [96]. Other approaches in clinical trials include immunotherapy, IDH1 inhibitors and DR5 agonists.

### Key recommendations

- Diagnosis of chondrosarcoma requires discussion in a bone sarcoma MDT.
- ACTs of the extremity can be observed initially. Curettage or excision can be considered for symptomatic or progressive lesions.
- Management of chondrosarcoma is surgical excision with wide margins.
- Radiotherapy may be helpful for treating unresectable disease and for palliation.
- Chemotherapy has a limited role in mesenchymal and dedifferentiated subtypes.

### Osteosarcoma

Adverse prognostic factors for conventional osteosarcoma include detectable metastases at presentation, axial or proximal extremity site, large tumour volume, raised serum alkaline phosphatase or LDH, older age, high body mass index (BMI), poor histological response to preoperative chemotherapy and pathological fracture [48, 97, 98]. Females may have better outcomes than males [98]. Multiple molecular prognostic biomarkers have been reported in the last few years, including circulating free and circulating tumour DNA (cfDNA and ctDNA,) [99], microRNAs and other small RNAs, specific circulating proteins, proteomic and transcriptomic profiles [100, 101]. Their role in standard care is not established.

### High grade osteosarcoma

Curative treatment for high-grade osteosarcoma comprises neoadjuvant chemotherapy, surgical resection and adjuvant chemotherapy, typically taking 6–9 months [102]. Combination treatment increases survival from 10–20% (surgery alone) to around 60% [103]. If possible, patients should enter clinical trials. For older patients, it is reasonable to consider surgery first [104], followed by adapted chemotherapy protocols [105].

Advantages of neoadjuvant treatment include rapid improvement of symptoms; early treatment of micrometastatic disease; facilitated resection in responsive tumours; time to plan primary surgery (e.g. manufacture customised endoprostheses); and prognostic information about the histological response, although a survival benefit over postoperative chemotherapy alone is unproven [106–108].

The most widespread regimen is multi-agent therapy with MAP (high-dose methotrexate (HDMTX), doxorubicin and cisplatin) and is recommended for UK patients with potentially resectable tumours up to 40 years of age (Table 2). Impaired renal function and certain drugs can delay methotrexate clearance, causing mucositis and nephrotoxicity and close monitoring is required.

**Table 2:** Systemic treatment for Osteosarcoma and other high-grade bone sarcomas*.

Category	1st Line	Subsequent therapy options
Patients < 40 years	Doxorubicin, cisplatin methotrexate (MAP) [114–116] +/−mifamurtide (approved in the UK for osteosarcoma patients < 30 years with localised, completed resected disease) [117]	Ifosfamide and etoposide [107] or tyrosine kinase inhibitor [140–142] ** or gemcitabine and docetaxel [138] or oral etoposide [139, 183]
Patients > 40 years, or intolerant to methotrexate	Doxorubicin and cisplatin [117]	

*Includes leiomyosarcoma, angiosarcoma, spindle cell sarcoma, dedifferentiated chondrosarcoma, malignant giant cell tumour.

**Compassionate-access in osteosarcoma.

For patients over 40 years and those who cannot tolerate HDMTX, regimens without methotrexate may still be effective [109, 110]. AP alone is considered suitable therapy, although doses are not standardised and may vary according to performance status, cardiac and renal function and other co-morbidities [109, 110].

The aim of surgery is complete tumour removal, preserving function where possible. Limb salvage is safe for most extremity tumours if adequate surgical margins can be achieved. Wide surgical margins reduce the risk of local recurrence but may not be possible. If there is a good histological necrosis rate ( > 90%) after chemotherapy, a closer surgical margin can be considered safe [111]. However, if there is a poor response to chemotherapy and “close” or positive margins, it is unclear whether amputation improves survival despite the increased risk of local recurrence [112].

The benefit of adjuvant chemotherapy over surgery alone was established many years ago [113] and long-term ( > 25 years) follow-up shows a significant survival benefit is maintained [114]. Adjuvant regimens may be the same as neoadjuvant or modified, but the ideal regimen and treatment duration for certain clinical situations remain undefined [115]. Changing adjuvant chemotherapy based on response has not improved outcomes and is not presently recommended [116]. For patients with overt progression on first-line chemotherapy; adjuvant therapy using ifosfamide and etoposide can be considered (Table 2).

Adding the immune modulator liposomal muramyl tripeptide (mifamurtide) to adjuvant chemotherapy demonstrated a statistically significant advantage in overall survival and a trend in event-free survival in a large, randomised trial [117] and has been approved in Europe for patients under 30 years with completely resected localised osteosarcoma, although the survival benefit in combination with MAP chemotherapy in the only randomised trial is unclear. Histological response to induction therapy is a robust prognostic indicator [118]. Clinical assessment of response to chemotherapy is usually only possible after several cycles of chemotherapy: changes in the size and ossification of the tumour do not reliably reflect response. Other investigational approaches include FDG-PET [119] and MRI using radiomic analysis [120].

Using haematopoietic growth factors to increase dose intensity has not consistently improved survival [103] but may limit the morbidity of myelosuppression. Prophylactic antibiotics are recommended for patients at risk of neutropenic sepsis [121] but care should be taken that the chosen antibiotic does not delay methotrexate excretion.

Adjuvant radiotherapy is not routinely recommended for limb osteosarcoma following complete resection as there is insufficient evidence of efficacy to improve local tumour control. Radiotherapy may be considered for those with inoperable, axial primary osteosarcomas to achieve local tumour control, or for selected patients with axial tumours in the adjuvant setting where there is a high risk of local recurrence and further surgery is felt to be unacceptable [72, 122, 123]. This is often best delivered with particle therapy (PBT or CIRT) to allow dose escalation close to critical normal structures or to reduce late effects, particularly in the paediatric and young adult population.

### Low-grade central, parosteal and periosteal osteosarcomas

Low-grade central, parosteal and periosteal osteosarcoma have lower metastatic potential, and complete surgical removal is recommended. Resection histology may show high-grade areas associated with poorer outcomes [124, 125]. Chemotherapy may be considered for these cases although evidence is limited [124–128].

### Craniofacial osteosarcoma

Jaw and other craniofacial osteosarcomas present specific management problems, especially for local control, and must be referred to a bone sarcoma MDT before surgery. The use of chemotherapy is not clearly defined but is considered a standard treatment option [129]. 18FDG PET is more reliable than standard imaging in evaluating response to neoadjuvant chemotherapy in craniofacial bone sarcomas and may correlate better with outcome than histological response [130]. Radiotherapy with techniques such as proton beam or intensity-modulated radiotherapy (IMRT) may be offered to primary tumours where surgery is not possible or would lead to significant morbidity. Similarly, adjuvant radiotherapy should be considered if surgical margins are close or involved, or there is a high risk of local recurrence and further surgery is not possible.

### Metastatic disease

Patients with metastatic osteosarcoma are a heterogeneous group and may be treated using the same regimens as for non-metastatic osteosarcoma, provided surgical resection of all disease sites is feasible [131]. Approximately 30% of patients with primary metastatic osteosarcoma and over 40% of those who achieve complete surgical remission become long-term survivors. For those with inoperable disease, the intensity and toxicity of therapy regimens need to be carefully balanced with the impact on quality of life.

### Recurrent disease

The prognosis for recurrent disease is poor, with long-term post-relapse survival of less than a third. Early relapse and distant non-lung metastases are associated with a poorer prognosis [132].

Treatment for recurrent osteosarcoma should include surgical resection if complete surgical clearance is possible. Complete removal of pulmonary metastases can lead to long-term survival [133, 134], particularly if there is a small number of metastases which respond to chemotherapy [135]. Over a third of patients with a second surgical remission survive beyond 5 years, and patients with multiple recurrences may be cured if they are resectable: repeated thoracotomies are often warranted [136]. If metastases are inoperable the disease is usually fatal.

There is no standard second-line chemotherapy regime for recurrent osteosarcoma [131]. In patients with inoperable metastases, chemotherapy is associated with limited prolongation of survival, but a positive benefit in operable disease has been observed [137]. The choice of agents may therefore consider the prior disease-free interval, the extent of disease and whether surgical resection is possible. Ifosfamide and etoposide are associated with the highest response rates [107, 138, 139] (Table 2). Multi-targeted tyrosine kinase inhibitors (MTKIs) including cabozantinib, regorafenib and lenvatinib have demonstrated single-agent activity in phase II clinical trials. [140–142] These agents are not available within the NHS infrastructure but may be available through compassionate-access schemes [140–142]. Clinical trials exploring their use in combination with chemotherapy and as maintenance therapy are ongoing. Gemcitabine and docetaxel and oral etoposide may offer effective palliation with limited toxicity [138, 139]. Radiotherapy may palliate inoperable sites [143].

### Key recommendations

- Treatment for osteosarcoma involves chemotherapy and surgery under the care of a specialist bone sarcoma MDT.
- Patients should be informed about relevant clinical trials and supported to enter them.
- First line standard treatment is MAP chemotherapy for patients under 40 years.
- Mifamurtide may be offered to patients under 30 years without metastases after surgery.
- The primary tumour should be resected with negative surgical margins where feasible.
- Adjuvant radiotherapy is not recommended routinely after surgery.
- To determine if a surgical resection is adequate, the response to chemotherapy and the surgical margin should be considered.
- If surgical removal is not possible, radiotherapy can be used to achieve local tumour control.
- Excision of pulmonary metastases if possible, may prolong survival.
- Recurrent disease should be resected, if possible, and both chemotherapy and MTKIs may have a role.

### Ewing sarcoma

The 5-year survival for Ewing sarcoma is <10% with surgery or radiotherapy alone. Multimodality treatment including chemotherapy improves 5-year survival to almost 80% in localised disease [144] and 20 to 40% in metastatic disease [145] and has improved in the last 3 decades [146]. Prognostic factors include axial location, tumour volume, raised serum LDH, older age ( > 15 years), a poor histological response to preoperative chemotherapy and incomplete or no surgery for local therapy [147, 148]. Patients should be offered recruitment to open trials if they are available.

### Localised disease

Current protocols usually comprise neoadjuvant induction chemotherapy, local therapy and consolidation therapy. The most active agents in common use are vincristine (V), doxorubicin (D), cyclophosphamide (C), ifosfamide (I) and etoposide (E) (Table 3) [144, 149, 150]. Greater treatment intensity is linked to better outcomes: a two-weekly interval-compressed VCD/IE induction was demonstrated to be more effective than the same regimen given three-weekly and VDC/IE induction followed by IE/VC consolidation has better outcomes than VIDE induction and VAI or VAC consolidation [151] and is now the preferred first-line treatment for all patients who are medically fit to receive it. For older patients and those unable to tolerate interval compressed VDC/IE, treatment may be considered 3-weekly or using attenuated doses of these agents.

**Table 3:** Systemic treatment summary for localised or metastatic Ewing Sarcoma.

1st Line	2nd Line	3rd Line and other
Compressed VDC/IE × 14 [151, 216]	High dose ifosfamide [217] or Cyclophosphamide and topotecan [181, 218] or Irinotecan and temozolomide [219] + /− High dose chemotherapy (busulphan/melphalan or treosulphan/melphalan) with peripheral blood stem cell rescue [185] N.B. Choice of 2nd line therapy will depend on patient/disease specific factors (e.g. if chemotherapy is being given with curative intent) and/or patient choice	Cyclophosphamide and topotecan [181, 218] or Irinotecan and temozolamide [220] or MTKI* [140, 188, 221] or Gemcitabine and docetaxel [222, 223] or Carboplatin and etoposide [224] or oral etoposide [183, 225]

*V* vincristine, *I* ifosfamide, *D* doxorubicin, *E* etoposide, *C* cyclophosphamide.

*Compassionate-access.

For patients with a poor response to VIDE induction or large tumours ( > 200 mls), high-dose busulphan-melphalan chemotherapy (BuMel HDT) with autologous stem cell rescue may be beneficial (Table 3) [152]. However, this does not appear advantageous for those with pulmonary metastases treated with standard chemotherapy and whole lung irradiation (WLI) and its utility following VDC/IE is not defined [153].

### Local treatment

Local treatment decisions are frequently complex and require discussion between the bone sarcoma MDT, the patient and often their family. Discussion at the National Ewing MDT is recommended [154].

Complete removal of the primary tumour (meaning the parts of all anatomical structures involved in the original tumour volume) provides optimal local control but is not always feasible, for example, because critical anatomical structures are involved. Radiotherapy should be considered in addition to surgery if there is a poor radiological or histological response to chemotherapy, the surgical margins are inadequate, the tumour is large or is in a high-risk area (e.g. pelvis) [147, 155, 156]. Tumour volume change can be seen on MRI, and reliably reflects chemotherapy response [157] particularly if late. FDG PET also reflects histological response [158].

Radiotherapy may be given before or after surgery or as a definitive treatment [159].

Relative indications for preoperative radiotherapy include poor radiological response to induction chemotherapy, expected marginal resection, or a technical advantage to preoperative administration (e.g. anatomical locations such as pelvis or rib where preoperative treatment allows better definition of tumour volume or a smaller treatment volume than postoperative treatment) [160].

Specific indications for postoperative radiotherapy include (taken from Euro-Ewing 2012 radiotherapy guidelines [149]).

- positive surgical margins with microscopic residual disease (R1 excision; < 1 mm or tumour up to edge of resection specimen) if further surgery to achieve negative margins is not possible
- positive surgical margins with macroscopic residual disease (R2 excision), if further surgery to achieve negative margins is not possible (this should be unusual)
- if all tissues involved by the pre-chemotherapy tumour volume have not been excised, even if resection margins are negative
- if there is a poor histological response ( ≤ 90% necrosis) to pre-operative chemotherapy, even if the resection margins are negative
- a displaced pathological fracture of bone at primary site (unless it is possible to excise all contaminated tissue)
- certain tumour sites where local control is judged to be more difficult to achieve e.g.:
  - Spine and paraspinal sites – in these sites excision is rarely complete, and is often intra-lesional
  - Pelvis and sacrum – in these sites it is frequently difficult or impossible to be sure that the entire pre-chemotherapy tumour volume has been excised
  - Rib tumours when presenting with a malignant pleural effusion

Definitive radiotherapy is frequently recommended for tumours judged to be inoperable, those in anatomic locations where complete removal would cause unacceptable morbidity (e.g. pelvic and sacral tumours), in patients at unacceptable risk of significant surgical complications, and if the prognosis is poor (e.g. widespread bone metastases) such that morbidity of surgery is not appropriate. Decision-making on local therapy, balancing between surgery and radiotherapy, is nuanced and should be individualised, after thorough MDT discussion considering all factors for individual patients.

Techniques such as intensity-modulated radiotherapy (IMRT) are generally used to ensure the delivery of an optimal radiotherapy dose [161]. particle therapy (PBT or CIRT) may be advantageous where there are local critical structures (e.g. spinal cord), and for younger patients having curative treatment to reduce the risk of radiation-induced malignancy [162]. Pelvic spacers can be used to keep the bowel out of the radiation field, facilitating higher doses and preventing long-term bowel toxicity, although the use of techniques such as IMRT and particle therapy has made use less common.

Fatal toxicity has followed high-dose large volume radiotherapy after BuMel HDT [163, 164]. Therefore, patients whose radiotherapy fields include critical organs such as the gut, spinal cord, brain or significant volumes of the lung (typically central axial tumours) should not be offered BuMel HDT unless the dose to critical organs is limited.

Relative contraindications to radiotherapy include:

- Impaired wound healing or biological reconstruction following surgery.
- Increased risk of infection of massive endoprostheses.
- Morbidity of radiotherapy in very young patients.
- Risk of radiation-induced malignancy.

### Metastatic disease

Around 26% of patients with Ewing sarcoma have metastatic disease at presentation (10% lung, 10% bone/bone marrow, 6% combinations or others) [165, 166]. Bone metastases confer poorer outcomes than lung/pleural metastases ( < 21% compared with 55% 5-year relapse-free survival) [167].

Systemic treatment for metastatic disease is similar to treatment for localised disease (Table 3). Several non-randomised and randomised trials have evaluated intensive, time-compressed or high-dose chemotherapy approaches.

For patients with pulmonary metastases, whole lung irradiation (WLI) following VDC/IE chemotherapy is indicated if the disease is not progressing on induction chemotherapy [168–170]. In two contemporaneous studies, BuMel high-dose therapy did not appear advantageous compared to chemotherapy plus WLI [153].

High-dose therapy with autologous stem cell rescue for patients with bone metastases and mixed metastatic disease has been evaluated in a non-randomised stratum of the Euro-EWING99 trial and as a randomised comparison with standard VIDE chemotherapy in the GPOH EWING 2008 trial [167, 171]. Neither trial demonstrated a clear benefit of high-dose therapy over historical controls (Euro-EWING99) or standard-dose chemotherapy (EWING 2008), although both suggested a possible advantage for patients under 14 years. The role of high-dose therapy in the context of interval-compressed VDC/IE induction chemotherapy is not established. Therefore, standard systemic therapy for high-risk disseminated disease remains interval-compressed VDC/IE chemotherapy.

Radiotherapy for bone metastases can provide palliation and local control [172]. Stereotactic body radiotherapy (SBRT) achieved an estimated local control rate of 85% in 27 bone or lung metastases in 14 patients with metastatic and recurrent Ewing sarcoma and osteosarcoma. However, there was significant toxicity, especially with concurrent chemotherapy and reirradiation [173]. In the GPOH cohort of patients recruited to the EURO-EWING 99 trial with widely disseminated disease, local treatment of both primary tumour and metastatic sites (most commonly with radiotherapy) was associated with better EFS, compared to those with local control to either primary site or metastatic sites, and to those with no local control, particularly in those with responsive metastatic disease [174].

The role of surgical resection of residual metastases is less well-defined. Patients with bone or bone marrow metastases and those with recurrent disease still fare poorly, with 5-year survival of between 10 and 45% [167, 175].

Patients with indeterminate pulmonary lesions have a good outcome and should be managed with curative intent [176].

### Recurrent disease

Recurrent disease is associated with poor outcomes [177]. However, patients relapsing more than 2 years after diagnosis and with isolated primary tumour recurrence have better outcomes than others [49, 178–180].

Although multiple chemotherapy regimens have been used in recurrent disease, the literature comprises multiple small patient series, non-standardised outcome measures and highly variable dosing regimens. The rEECur trial has compared the four most used drug combinations in a multi-arm, multi-stage randomised phase II/III trial. Multiple pairwise comparisons between arms have defined the following hierarchy based on EFS, OS and RECIST 1.1 imaging response after four cycles of chemotherapy, in order of decreasing efficacy: high dose ifosfamide, topotecan and cyclophosphamide [181], irinotecan and temozolomide, and gemcitabine and docetaxel [182]. The difference in absolute EFS and OS between topotecan/cyclophosphamide and irinotecan/temozolomide is small and there are significant differences in the toxicity profiles of each regimen, with a preponderance of myelotoxicity and neutropenic fever with ifosfamide and topotecan/cyclophosphamide, a small rate of significant encephalopathy and renal toxicity with ifosfamide and gastrointestinal toxicity with irinotecan/temozolomide. Oral etoposide is also frequently used to treat recurrent Ewing sarcoma. The evidence base is poor. A recent analysis of its use in childhood and young adulthood in the UK demonstrated poor survival [183]. There have been no randomised trials of oral etoposide.

High-dose chemotherapy followed by autologous stem cell rescue has not been evaluated in a randomised trial. Several observational studies suggest a benefit in selected patients; it may be considered as consolidation therapy in the context of no or minimal residual disease, but its use remains controversial [184, 185].

Local control with radiotherapy and/or surgery may be helpful to palliate local symptoms but the contribution to long-term disease control has not been robustly evaluated.

Several molecularly targeted agents have been tested in relapsed Ewing sarcoma. The most promising in terms of activity, toxicity and availability are the multi-targeted tyrosine kinase inhibitors [186]. Pazopanib, cabozantinib and regorafenib have been reported to show single-agent activity [140, 141, 187, 188]. The sequencing of chemotherapy and TKIs, and whether there is any benefit in adding TKIs as maintenance therapy following chemotherapy has not been evaluated in relapsed disease. Decision-making in the absence of evidence of benefit must be balanced with potential toxicity and the need for repeated hospital visits in a disease setting where the median OS is approximately one year.

Consideration should be given to enroling all patients with relapsed disease in clinical trials where possible.

### Key recommendations

- For Ewing sarcoma, systemic treatment with chemotherapy is standard. VDC/IE regime has demonstrated superiority over VIDE.
- Local treatment decisions are complex requiring thorough MDT discussion, and presentation at the UK, National Ewing Multidisciplinary Team meeting is recommended.
- When treating the primary tumour with curative intent, all structures involved in the pre-chemotherapy volume should be treated with surgery, radiotherapy or both.
- If radiotherapy is indicated this can be delivered pre- or post-operatively or as definitive treatment.
- Patients with newly diagnosed and relapsed disease should be considered for clinical trials.

### Other round cell sarcoma including BCOR-altered and CIC-rearranged tumours

These tumours are now accepted as distinct entities. Combination treatments with chemotherapy should be considered although optimal therapy is not defined. These sarcomas are commonly treated using Ewing sarcoma protocols. While outcomes in patients with BCOR-altered sarcoma are comparable with those in Ewing sarcoma, survival with CIC-rearranged sarcoma is poor irrespective of chemotherapy; use of soft tissue sarcoma regimens such doxorubicin and ifosfamide have similar outcomes for those with localised disease. No treatment at relapse has been found to be of benefit [20, 21]. Patients should be entered into clinical trials if possible [20, 21].

### Chordoma

Assessment in a specialist centre with expertise in managing chordomas is essential. En bloc resection with a margin of 1 mm or more is the recommended treatment where technically feasible and the sequelae of surgery are accepted by the patient [4, 24]. In the sacrum, surgery is the recommended treatment for tumours involving the S4 nerve root or below. Above this, surgical morbidity increases and therefore should be discussed in the context of other treatments, including radiotherapy. Sacrectomy procedures are technically demanding with a high risk of complications and require access to the appropriate surgical expertise, including sarcoma, spinal and plastic surgeons as appropriate.

Tumours of the skull base or cervical spine should be removed as completely as possible, whilst preserving neurological function and therefore quality of life. R0 resection is rarely possible. Eight studies (summarised by Stacchiotti et al. [24]) showed surgery (R1 and R2 resections) followed by radiotherapy in selected patients produced 5-year estimated overall survival of 55–86% for chordoma of the skull base and/or cervical spine [24].

High-dose adjuvant radiotherapy is beneficial after surgery with positive or close surgical margins [24, 189]. Proton beam therapy or carbon ion radiotherapy are promising alternatives, particularly for high sacral tumours where surgical morbidity is high [190, 191].

Metastases are rare but local recurrence is common and difficult to cure [192]. Treatment for local recurrence may include surgery and/or radiation therapy and/or systemic treatment [4, 192]. Local treatment such as surgery, radiofrequency ablation, cryotherapy or stereotactic radiotherapy should be considered for oligometastatic disease in selected cases.

There is no standard of care systemic therapy for patients with advanced/ metastatic disease. Imatinib is commonly used, but evidence is limited, and it is not currently commissioned for use within the NHS so is not uniformly available [193]. EGFR inhibitors have shown potential benefit in small retrospective series and may be available through compassionate-access schemes, but additional evidence is required for to be accepted as standard of care. The results of the first prospective international trial evaluating afatinib in chordoma in this setting are eagerly awaited [194]. Immune-checkpoint inhibitors too show promise in retrospective single centre series [195]. Prospective studies are warranted to further evaluate efficacy. Patients should be recruited to clinical trials wherever possible.

### Other high grade malignant bone sarcomas

These include undifferentiated pleomorphic sarcoma of bone and spindle cell sarcoma of bone which is a diagnosis of exclusion. Prognosis and prognostic factors are similar to those of patients with osteosarcoma and treatment should follow similar protocols [33, 196, 197] (Table 2).

Adamantinoma is a malignant tumour occurring in the tibia. Most are low grade but higher-grade areas in the primary tumour may require systemic therapy. Complete excision is the treatment of choice [198].

### Giant cell tumour of bone

All patients with GCTB should be managed by a bone sarcoma MDT. Brown tumours of hyperparathyroidism should be excluded with serum calcium levels. There are few prospective, randomised clinical trials, but large single-institution series have led to consensus about prognosis and management.

Surgery is the treatment of choice for resectable GCTB. En-bloc excision is associated with lower rates of recurrence than intralesional curettage. However, curettage usually preserves more function. Local control is improved with surgical adjuvants such as high-speed burring, cement and cryotherapy [199]. The choice of surgical approach depends on whether joint preservation is possible, and the size of any soft tissue mass and should weigh morbidity of treatment against the risk of recurrence.

Denosumab is a fully human monoclonal antibody to RANKL, suppressing the formation and activity of osteoclasts. In a proof-of principle phase II study of 35 patients with recurrent or unresectable GCTB, 30 patients (86%; 95% confidence interval 70–95) had a tumour response, showing near complete elimination of giant cells (20 of 20 evaluable patients) or radiological stabilisation of disease at 6 months (10 of 15 evaluable patients). 26 of 31 evaluable patients reported reduced pain or improvement in functional status and nine demonstrated bone repair. Response was usually associated with rapid changes in avidity on PET scan and suppression of bone turnover (reduced urinary N-telopeptide and serum C-telopeptide) 28 days after the first dose and sustained for the study duration [200]. The FDA and subsequently EMA (13/07/2011) granted marketing authorisation and conditions for use of Denosumab in GCTB.

Denosumab is indicated where surgery is not possible or unacceptably morbid and in patients with metastases. It is also used for selected cases before surgery to solidify the soft tissue component, facilitating surgical resection and reducing the risk of recurrence. Curettage after denosumab can be difficult and is associated with a higher risk of local recurrence [201]. Therefore, complete resection is usually preferred after denosumab treatment.

Denosumab is given as a monthly subcutaneous injection after three loading doses at weekly intervals. All patients require daily calcium and vitamin D supplements and must avoid pregnancy by using adequate contraception. Significant side effects include hypocalcaemia, osteonecrosis of the jaw and atypical fractures [202]. Whilst initial control is excellent (96%), most tumours recur if the drug is stopped (after around 9 months), so surgical resection is indicated where possible. The optimal duration of pre-operative treatment is not clear, but treatment for up to 6 months is reasonable for responding tumours. Inoperable tumours may require life-long treatment but the consequences of this, particularly in younger patients, are not known [203].

Radiotherapy has been used historically where surgery was judged to be unacceptably morbid or adequate margins were difficult to achieve. Local control rates approach 80% but are lower in heavily pre-treated patients [204].

Cytotoxic chemotherapy has been used in unresectable advanced GCTB not responding to denosumab, but there are no randomised clinical trials, and chemotherapy is not standard of care.

### Treatment of metastatic disease

Patients with metastatic disease may require life-long treatment with denosumab. Retrospective studies have increased the interval between doses in patients with stable disease two years after starting treatment from 4 weekly to 8 weekly [205]. Surgery for pulmonary metastases is usually not performed.

### Malignant giant cell tumours of bone

Rarely, GCTs can transform to or present as malignant high-grade tumours. Patients do not benefit from denosumab. Combination cytotoxic chemotherapy following protocols for osteosarcoma and other high-grade PMBT should be considered (Table 2).

## Follow-up and survivorship

Follow-up after treatment aims to detect local and systemic recurrence, manage long-term toxicity of chemotherapy and radiotherapy and the complications of surgery. Local recurrences are often detected by patients and therefore information about what to do if local recurrence is suspected should be provided.

Clinical follow-up of patients treated for high-grade tumours should include physical examination of the primary tumour site, and assessment of the functional outcome and possible complications of any reconstruction. Local and chest imaging should be included. Evidence for the optimum frequency of follow-up and the best imaging investigations is lacking although a randomised controlled trial showed no benefit of greater frequency of follow-up with regular cross-sectional imaging over standard follow-up [206].

Current protocols recommend follow-up at intervals of 2–4 months for the first 3 years after completion of therapy, every 6 months for years 4 and 5 and thereafter annually [207, 208]. Modelling of metastatic events suggests chest surveillance annually to 5 years for low-grade sarcomas, every 3 months for 2 years then annually to ten years for intermediate-grade sarcomas, and every 3 months for 2 years, every 6 months from 2 to 5 years and annually from years 5 to 10 for high-grade sarcomas [209].

For low-grade bone sarcomas, the frequency of follow-up visits can be reduced to 4–6 monthly for 2 years and then annually. Late metastases as well as local recurrences and failure of reconstructions may occur more than 10 years after diagnosis in all tumours and there is no universally accepted stopping point for follow-up.

Although evidence for local site imaging is lacking, for chordoma the high risk of occult local recurrence warrants MRI of the primary site at 6 months, 1 year and then annually to ten years. Similarly, in patients at high risk of occult local recurrence such as after resection of pelvic chondrosarcoma, regular MRI of the primary site may be reasonable. Plain X-rays of the local site are standard to detect radiological local recurrence and potential complications of the surgical reconstruction.

It is important to evaluate the long-term toxicity of chemotherapy and radiotherapy as well as immediate chemotherapy-related complications [210]. Monitoring for late effects should be undertaken, depending on the treatment and in conjunction with available late effect services [211, 212].

Secondary cancers may arise in survivors of bone sarcomas, either related to or independent of treatment. Secondary leukaemia (particularly acute myeloid leukaemia) may rarely occur as early as 2–5 years after chemotherapy [213, 214]. The increasing use of molecular profiling, including WGS for all sarcoma patients in England, is expected to increase the proportion of patients with identified pathological germline cancer predisposition syndromes. Where relevant, for instance in patients with osteosarcoma occurring in the setting of Li-Fraumeni syndrome, appropriate cancer surveillance programmes should be followed [215].

### Key recommendations

- Standard follow-up for all sarcoma cases is chest X-ray, local site x-ray and clinical review.
- It is reasonable to consider MRI of the local site regularly for sacral chordoma and other sites at risk of occult local recurrence, such as the pelvis.
- At the end of treatment, patients should receive information about the risk of local and systemic recurrence.
- Patients should have access to services for the late effects of treatment including chemotherapy, radiotherapy, surgery and psychosocial support.
